# Probiotic bacteria of wild boar origin intended for piglets – An *in vitro* study

**DOI:** 10.17221/35/2024-VETMED

**Published:** 2024-08-29

**Authors:** Iveta Kostovova, Katerina Kavanova, Monika Moravkova, Jan Gebauer, Lenka Leva, Monika Vicenova, Vladimir Babak, Martin Faldyna, Magdalena Crhanova

**Affiliations:** ^1^Department of Microbiology and Antimicrobial Resistance, Veterinary Research Institute, Brno, Czech Republic; ^2^Department of Infectious Diseases and Preventive Medicine, Veterinary Research Institute, Brno, Czech Republic

**Keywords:** antibiotic susceptibility, antimicrobial activity, bacteriocins, exopolysaccharides, interleukin-10

## Abstract

Using probiotics represents a potential solution to post-weaning diarrheal diseases in piglets on commercial farms. The gastrointestinal tract of wild boars serves as a promising reservoir of novel lactic acid bacteria with suitable probiotic characteristics. In this study, we isolated eight bacterial strains from the intestinal content of wild boars identified as representatives of the species *Bifidobacterium apri*, *Lactobacillus amylovorus*, and *Ligilactobacillus salivarius*. These isolates underwent *in vitro* analysis and characterisation to assess their biological safety and probiotic properties. Analysis of their full genome sequences revealed the absence of horizontally transferrable genes for antibiotic resistance. However, seven out of eight isolates harboured genes encoding various types of bacteriocins in their genomes, and bacteriocin production was further confirmed by mass spectrometry analysis. Most of the tested strains demonstrated the ability to inhibit the growth of selected pathogenic bacteria, produce exopolysaccharides, and stimulate the expression of interleukin-10 in porcine macrophages. These characteristics deem the isolates characterised in this study as potential candidates for use as probiotics for piglets during the post-weaning period.

Weaning is a critical stage in porcine production that presents significant challenges for farmers and can lead to substantial economic losses. During this stage, the piglets are exposed to various stressors, including separation from the sow and littermates, transportation, handling, transition from milk to solid pelleted feed, and housing with piglets from other litters (Hwang et al. 2016; Shin et al. 2019). These changes collectively influence the health of piglets resulting in greater susceptibility to pathogen infection manifested in diarrhoea, transient anorexia, reduced feed conversion efficiency, loss of weight, and, in extreme cases, death.

Antibiotic feed additives were commonly used preventively to mitigate losses associated with weaning. However, the increasing risk of spreading antimicrobial resistance among pathogenic bacteria has reduced the use of antimicrobial drugs in farm animals (Tang et al. 2022). In addition, the prophylactic use of antibiotics and their use as growth promoters have been banned in the European Union (EU) since 2006. In 2022, the EU implemented legislation restricting the prophylactic and metaphylactic use of antimicrobials in animals to only exceptional cases or instances of high-risk spread of infectious diseases, intended to reduce the overall proportion of antimicrobial use in animals (EP 2019; ECDC et al. 2021).

Therefore, there is a pressing need for an alternative to antibiotics to prevent post-weaning diarrhoea in piglets (McEwen and Collignon 2018). One promising solution is represented by probiotics, defined as microorganisms (bacteria or yeasts) that positively affect the host when administered in sufficient quantities. Lactic acid bacteria (LAB), primarily lactobacilli, and bifidobacteria are the most commonly used probiotics (Shin et al. 2019). The beneficial effects of lactic acid bacteria on the host organism include enhanced immune function, inhibition of the adhesion of pathogens to the epithelial surface, and improved digestion connected to their ability to produce lactic acid and metabolites such as antioxidants, organic acids, and antimicrobial compounds (Azad et al. 2018). This modulation improves intestinal microbial balance (Li et al. 2020; Zhou et al. 2020).

The compounds produced by LAB that inhibit pathogen growth include hydrogen peroxide, diacetyl, organic acids, and bacteriocins. Bacteriocins are antimicrobial peptides that inhibit or kill pathogenic bacteria in the host gut and alter the gut microbiome composition in animal models (Anjana and Tiwari 2022). The production of bacteriocins is, therefore, one of the most crucial parameters for selecting probiotic strains as an alternative to antibiotics. Additionally, the production of exopolysaccharides (EPs) by LAB is another important parameter for probiotic selection. EPs produced by LAB are reported to have antimicrobial, immunomodulatory, anti-inflammatory, antioxidant, anti-tumour, anti-viral, anti-diabetic, anti-ulcer, and cholesterol-lowering properties in humans (Angelin and Kavitha 2020).

Various sources including cereals, fruits and vegetables, and dairy-based products such as milk are typically used to isolate and select potential probiotic strains. However, in addition to feed and food sources, the gastrointestinal tract (GIT) of healthy individuals can also serve as a feasible isolation source (Guo et al. 2010). In contrast to domestic pigs, which are frequently exposed to antibiotics, the GIT of wild boars presents a promising reservoir of potentially probiotic strains. Wild boars inhabit forest areas and must adapt to natural resources, exhibiting strong adaptability to their ecological conditions. Unlike domestic pigs, wild boars do not rely on veterinary antimicrobials for survival, making them a promising reservoir of health-promoting LAB (Li et al. 2020).

Between 2019 and 2021, we successfully isolated more than 60 strains of LAB and bifidobacteria from the GIT of wild boars hunted in the Czech Republic. Our initial analysis focused on ensuring the safety of these isolates, which involved various measures such as antimicrobial susceptibility testing, evaluation of haemolysin production and wholegenome sequencing to detect horizontally transmissible genes associated with antibiotic resistance. Subsequently, we selected eight isolates considered “safe” and investigated their potential for EP production. Ultimately, we tested these eight isolates to determine their capability to inhibit the growth of pathogenic bacterial strains (enteropathogenic *Escherichia coli*, *Salmonella* Typhimurium, and *Yersinia enterocolitica*). Those isolates were identified as *Bifidobacterium apri* (*B. apri*) (115B), *Lactobacillus amylovorus* (*L. amylovorus*) (M597AA, M597B, M624A, M668A, M696A), and *Ligilactobacillus salivarius* (*L. salivarius*) (M494A, M698A).

This study aimed to assess *in vitro* the impact of these eight selected strains on two crucial parameters for selecting efficient probiotics: the production of antibacterial substances and the modulation of the immune system. A combination of molecular techniques was employed to detect the presence of genes for antibacterial peptides and confirm their expression at the protein level. A model of macrophages derived from monocytes was also utilised to evaluate the modulation of immune cells. These cells were stimulated with pro-inflammatory lipopolysaccharide to mimic an inflammatory response.

## MATERIAL AND METHODS

### Isolation of bacterial strains and growth conditions

All strains used in this study were isolated from the digestive tract of wild boars from various locations in the Czech Republic. Samples from the small and large intestines were collected during wild boar hunts conducted in 2018 and 2019. A total of 42 digestive tract samples from wild boars were collected and immediately placed in coolers to maintain their integrity. The samples were cultured on retrieval on Rogosa agar (Oxoid, Basingstoke, UK). Cultivation was carried out simultaneously under anaerobic conditions (using anaerobic jars with palladium catalysts maintaining an atmosphere of 10% CO_2_/10% H_2_/80% N_2_) and microaerophilic conditions at 37 °C. Subsequently, isolates were sub-cultured on de Mann Rogosa Sharpe agar (MRS; Oxoid) under anaerobic conditions at 37 °C for 48 hours. More than 60 strains of *Lactobacillus* and *Bifidobacterium* were isolated from the intestines of wild boars during the study.

### DNA isolation and whole-genome sequencing

All strains underwent genomic DNA extraction using a Quick-DNA^TM^ Faecal/Soil Microbe Microprep Kit (Zymo Research, Irvine, CA, USA) following the manufacturer’s instructions. The extracted DNA was utilised for library construction using the Nextera Library preparation kit. Paired-end sequencing was conducted using the NextSeq platform, employing a NextSeq 500/550 High Output Kit v2.5 from Illumina (Illumina Inc., San Diego, CA, USA). The generated read sequences underwent trimming using Trim Galore v0.6.7 (accessed on 1 December 2020), and low-quality reads were eliminated using Cutadapt v0.6.6. Following the removal of low-quality reads, MultiQC v1.9 was employed to evaluate the quality of the remaining reads. The trimmed reads were subsequently subjected to *de novo* genome assembly using Unicycler v0.4.9b, which utilised SPAdes v3.14.1.

### Strain identification

The individual isolates were identified using sequencing analysis of the 16S rRNA gene using the primers 16S27f (AGAGTTTGATCMTGGCTCAG) and 16S1492r (TACGGYTACCTT-GTTACGACTT) (Lagace et al. 2004). Subsequently, the PCR products were purified using a QIAquick PCR Purification Kit (Qiagen, Valencia, CA, USA). The resulting amplicons were sequenced in both the forward and reverse directions using a Mix2Seq Kit by Eurofins Genomics (Luxembourg City, Luxembourg). The isolated bacterial strains were identified based on sequence similarity with reference sequences in the GenBank and EzBioCloud databases accessed on 1 October 2020.

The final identification of the isolates relied on the average nucleotide identity (ANI) of all orthologous genes shared between the genome of the type strain and the genome of the particular isolate. ANI calculation was performed using the bioinformatics tool FastANI. An isolate was considered to belong to a particular species if the ANI value between the type strain genome and the genome of the isolate exceeded 95%. The genome sequences of the following type strains were used: *Bifidobacterium apri* DSM 100238, *Ligilactobacillus salivarius* DSM 20555, and *Lactobacillus amylovorus* DSM 20531.

### Analysis of antibiotic resistance and bacteriocin genes

The presence of horizontally acquired antibiotic resistance genes was detected using whole-genome sequencing data. Bacterial genomes were analysed using Abricate v1.0.1 software with the following databases: Comprehensive Antibiotic Resistance Database (CARD), ResFinder, Argannot, Megares, and NCBI AMRFinderPlus. All databases were updated on 7 February 2022 (Moravkova et al. 2022).

The genes potentially responsible for bacteriocin production were identified using the web-server BAGEL4.

### Antimicrobial susceptibility

Antimicrobial susceptibility testing was conducted using broth microdilution methods in accordance with ISO10932:2010 standards and the interpretation criteria suggested by EFSA FEEDAP Panel guidance (EFSA et al. 2018). The microplates were incubated for 48 h at 37 °C in an anaerobic atmosphere. The minimal inhibitory concentration (MIC) was visually read as the lowest concentration of the antimicrobial substance that inhibited bacterial growth. The following antimicrobials were tested: ampicillin (0.125–16 mg/l), streptomycin (2–256 mg/l), tetracycline (0.5–64 mg/l), erythromycin (0.063–8 mg/l), clindamycin (0.063–8 mg/l), chloramphenicol (0.25–32 mg/l), kanamycin (0.5–2050 mg/l), gentamicin (0.125–512 mg/l), vancomycin (0.25–32 mg/l) and ciprofloxacin (0.125–128 mg/l). All tested antimicrobials were purchased from Sigma-Aldrich (St. Louis, MO, USA). Quality control strains (*Lactobacillus plantarum* ATCC14917 and *Lactobacillus paracasei* ATCC334) were used to ensure the accuracy of susceptibility testing (Moravkova et al. 2022). The evaluation of susceptibility was based on microbiological cut-off values established by the EFSA for the *Lactobacillus acidophilus* group (used for *L. amylovorus* strains), *Lactobacillus* facultative heterofermentative (used for *Ligilactobacillus salivarius*) and *Bifidobacterium* (used for *Bifidobacterium apri*).

### β-haemolysin production

To cultivate the bacterial isolates, MRS agar medium supplemented with cysteine at a concentration of 0.3 g/l (MRS+C) was utilised and incubated for 48 hours. The bacterial cultures were then mixed with a physiological solution to prepare a suspension with a 1.2 McFarland turbidity. Columbia agar with 5% sheep blood (Oxoid, Basingstoke, UK) was employed to observe the production of β-haemolysin. Five microliters of each bacterial suspension were spotted in triplicate on the surface of the Columbia agar plate. The plates were then incubated for 48 h at 37 °C under anaerobic conditions.

### Antimicrobial activity

The antimicrobial activity of all bacterial isolates was assessed against pathogenic bacteria known to cause diarrhoeal infections, including three isolates of *Escherichia coli* (EC 971, EC973, EC974) producing enterotoxins, one isolate of *Salmonella* Typhimurium (STM 970) and one isolate of *Yersinia enterocolitica* (YE M108/15), all originating from the gastrointestinal tract of pigs. The agar spot test described by Monteiro et al. (2019) was performed with some modifications. Five μl of *L. salivarius* suspension in physiological solution (McFarland turbidity 1.3) was spotted on 15 ml of MRS agar plated in a Petri dish. After 24 h of incubation at 37 °C under anaerobic conditions, 10 ml of tryptic soy agar (TSA, HiMedia, Brno, Czech Republic) was overlaid onto the MRS agar containing the grown culture of a particular tested isolate. The TSA medium was allowed to solidify at room temperature; thereafter suspensions of pathogenic bacteria (McFarland turbidity 0.5) were spread with a swab. The plates were then incubated at 37 °C for 24 h under aerobic conditions. The indication of antimicrobial activity was the formation of a clear halo around a grown probiotic culture spot. The diameter of the growth inhibition halo was measured and expressed in millimetres.

### Production of exopolysaccharides

The ability to produce exopolysaccharides (EPs) was examined by picking the colonies growing on the surface of MRS agar plates with a sterile bacteriological loop and observing the formation of a filament when the loop was lifted (Ruas-Madiedo and de los Reyes-Gavilan 2005).

### Preparation of supernatants for *in vitro* assays

All selected isolates were cultivated on MRS+C agar for 48 h at 37 °C. Subsequently, one bacteriological loop of the culture was transferred into 1 ml of MRS+C medium and incubated for 24 h at 37 °C. Following this, 500 μl of the suspension was transferred into 50 ml of MRS+C medium. All cultivation steps were conducted under anaerobic conditions. Once the bacterial cultures reached the beginning of the stationary phase, the bacterial cells were collected by centrifugation at 2 700 RCF for 15 minutes. The resulting supernatant was adjusted to pH 7 with 5 M NaOH. Cells were washed three times in 9.5 mM (PO_4_) Dulbecco’s Phosphate Buffered Saline with Calcium and Magnesium (DPBS; Lonza, Basel, Switzerland) and then resuspended in DPBS to a concentration of 4–5 × 10^8^ CFU/ml.

### Mass spectrometry analysis of secreted proteins

Supernatants obtained from bacterial cultures (as described in the previous paragraph) were collected and a protein concentration was estimated by UV280 measurement using a DeNovix DS-11 FX spectrophotometer (DeNovix, Wilmington, DE, USA) with bovine serum albumin as a calibrator. Ten μg of total protein was utilised for mass spectrometry (MS) sample preparation with the filter-aided sample preparation (FASP) method (Wisniewski et al. 2009). Each sample underwent six washes with 8 M urea in Vivacon 500 centrifugal tubes (Sartorius Stedim, Göttingen, Germany) equipped with a 10 000 MWCO membrane filter. Dithiothreitol (10 mM, Sigma-Aldrich) and iodoacetamide (50 mM, Serva, Heidelberg, Germany) in 25 mM TEAB (triethylammonium bicarbonate; Sigma-Aldrich) buffer were used for reduction and alkylation, respectively. The proteins were then digested with trypsin (Thermo Fischer Scientific, Waltham, MA, USA) at a 1 : 50 ratio, initially for one hour at 37 °C followed by overnight digestion at 25 °C. Following centrifugation, the eluate containing digested peptides was evaporated using a DNA120 SpeedVac (Thermo Fischer Scientific), and the peptide pellet was resuspended in 0.1% aqueous formic acid (Sigma-Aldrich) serving as the mobile phase for liquid chromatography (UltiMate 3000 RSLCnano; Thermo Fischer Scientific). Peptides were separated and eluted using a 2-hour gradient with increasing concentrations of acetonitrile (0.1% formic acid in 80% acetonitrile; Sigma-Aldrich, St. Louis, MO, USA) at a flow rate of 300 nl/min. Separation of the peptides was performed on a 25 cm column (Acclaim PepMap RSLC C18, 2 μm, 100 Å, 75 μm I.D.; Thermo Fischer Scientific) with the uHPLC system connected to an EASY-spray ion source and a Q Exactive mass spectrometer (Thermo Fischer Scientific). A survey scan over the m/z range 390–1 700 was conducted to identify protonated peptides with charge states of at least 2, which were subsequently selected for data-dependent MS/MS analysis and fragmented by HCD dissociation. Ten fragment mass spectra following each full scan were recorded. The measured spectra were then searched using Proteome Discoverer (v2.4; Thermo Fischer Scientific) with Sequest HT as a searching algorithm. Uniprot unreviewed databases for the *Bifidobacteriales* taxon (from 2022/08) and *Lactobacillales* taxon (from 2022/08) were employed in Sequest HT. Peptides with a false discovery rate of less than 0.01 were considered well-identified. The quantity of identified proteins was expressed by the intensity of the chromatographic peak detected by the mass spectrometer. The summed abundances of the connected peptide groups provided the quantification of each identified protein. Relative abundance was calculated after normalization for the total peptide amount in each sample and scaled to a value of 100, representing the median abundance.

### Isolation of monocyte-derived macrophages

The preparation of monocyte-derived macrophages (MDMs) followed previously described methods (Kavanova et al. 2017). CD14^+^ porcine monocytes were isolated from heparinised peripheral blood obtained from five-month-old pigs. Mononuclear cells were isolated using Histopaque-1077 (Sigma-Aldrich) gradient. Monocytes were further enriched to a purity of > 95% using positive magnetic bead selection (QuadroMACS^TM^ cell separator; Miltenyi Biotec, Bergisch Gladbach, Germany) with a monoclonal antibody directed against CD14 (clone MIL2, AbD Serotec, 1 μl per 10^8^ cells) and goat anti-mouse IgG microbeads along with LS separation columns (MACS).

After isolation, the cells were washed with Dulbecco’s Modified Eagle’s Medium (DMEM; Gibco, New York, USA), centrifuged at 1 100 × *g* at 20 °C for 10 min, and resuspended with DMEM supplemented with 10% Fetal Bovine Serum Superb (FBS; Diagnovum, Tillburg, The Netherlands) and 1% antibiotics (Antibiotic Antimycotic Solution 100 ×: 10 000 units penicillin, 10 mg streptomycin, and 25 μg amphotericin B per ml; Sigma-Aldrich). MDM were then plated into 24-well culture plates (Biotech) at a concentration of 5 × 10^5^ cells in one ml per well or, in the case of the viability/cytotoxicity assay, into black Nunc-Immuno^TM^ MicroWell^TM^ 96-well polystyrene plates (Sigma-Aldrich) at a concentration of 1 × 10^5^ cells at 0.2 ml per well. These cells were then incubated for 5 days at 37 °C, 5% CO_2_.

After 5 days of transformation into MDM, the DMEM medium was removed and the cells were washed in DPBS. Alternatively, the medium was enriched with either 10% of supernatants or washed bacteria at a ratio of 1: 10. The cells were either left unstimulated or stimulated with LPS (1 μg/ml; lipopolysaccharides from *E. coli* O111:B4; Sigma-Aldrich) for 6 hours.

The analysis of mRNA expression based on reverse transcription qPCR was subsequently performed.

### Gene expression analysis based on reverse transcription quantitative real-time PCR (RT-qPCR)

The mRNA expression of the anti-inflammatory cytokine interleukin-10 (IL-10) in MDM was determined using RT-qPCR. Following treatments, MDMs were stabilised in TRI Reagent RT (Molecular Research Center, Cincinnati, OH, USA) and stored at –70 °C until RNA isolation. The RNA phase was obtained from the mixture with bromanisole by separation in a refrigerated centrifuge. Total RNA was isolated using a NucleoSpin RNA Mini Kit (Macherey Nagel, Düren, Germany) according to the manufacturer’s instructions, resulting in a final volume of 36 μl RNeasy free water. The purity and integrity of RNA were assessed spectrophotometrically by measuring absorbance ratios at 230, 260, and 280 nm and by agarose gel electrophoresis. Reverse transcription was performed using M-MLV reverse transcriptase (200 IU/μl, Invitrogen) and oligo(dT) RT primer (Generi Biotech, Hradec Kralove, Czech Republic) at 37 °C for 1.5 hours.

Duplicates of 3 μl qPCR reaction were dispensed using a Nanodrop II liquid dispenser (Innovadyne Technologies, Rohnert Park, CA, USA) and qPCR was performed using a LightCycler 480 instrument (Roche, Basel, Switzerland). The reaction conditions included denaturation at 95 °C for 15 min followed by amplification in 45 cycles of 95 °C for 15 s and 58 °C for 30 s and elongation at 72 °C for 30 s according to the manufacturer’s recommendations. Quantification cycle data with a variation of less than 0.5 were further analysed. Each reaction contained 10 pmol of each primer pair (Generi Biotech), 1.5 μl of QuantiTect SYBR Green PCR MasterMix (Qiagen), and 0.5 μl of 4 × diluted cDNA. Analysis of the melting temperature confirmed the specificity of amplicons using LightCycler 480 1.5.0.39 software (Roche Applied Science, https://www.roche.com). Gene-specific primers for IL-10 were adapted from Kyrova et al. (2012). The reference housekeeping gene (REF) TBP1 (Nygard et al. 2007) was determined using a variability test (Andersen et al. 2004) among the MDM samples tested. Assuming a primer efficiency ≥1.9, normalised gene expression based on quantification cycle (Cq) values was calculated as 2^–(CqGENE – CqREF)^ (Livak and Schmittgen 2001; Bustin et al. 2009). REF served as a qPCR positive control. The data obtained were logarithmised and further analysed using two-factor analysis of variance in STATISTICA v13.2 (StatSoft Inc., Tulsa, OK, USA).

## RESULTS

### *De novo* assembly

*De novo* genome assembly was carried out on the genomes of one *Bifidobacterium apri*, five *Lactobacillus amylovorus*, and two *Ligilactobacillus salivarius* strains. The number of assembled contigs varied from 59 to 126, with L50 and N50 values ranging from 36 990 to 172 888 bp and 4 to 16 contigs, respectively. The genome sizes ranged from 1.9 to 2.3 Mbp with an average GC content of 37–38% in the *L. amylovorus* and *L. salivarius* strains and 59% in the *B. apri* strain (Table 1). ANI values were calculated against three type strain genomes: *B. apri* DSM 100238, *L. amylovorus* DSM 20531, and *Ligilactobacillus salivarius* DSM 20555. The ANI values of all used genomes and their respective type genomes exceeded the recommended 95% threshold for species delineation. Antimicrobial susceptibility, presence of antibiotic resistance genes and β-haemolysis.

**Table 1:** The genome assembly and ANI results-based strain identification

Isolate name	NCBI accession	Number of contigs	Contig size (bp)	L50 (contigs)	N50 (bp)	GC (%)	ANI (%)	Type strain with the highest similarity
115B	SAMN35847917	59	2 396 385	8	78 681	59.34	99.98	*Bifidobacterium apri* DSM 100238
M597AA	SAMN31135173	126	2 098 617	12	56 554	37.78	98.73	*Lactobacillus amylovorus* DSM 20531
M597B	SAMN31135174	122	2 095 652	13	56 022	37.78	98.65	*Lactobacillus amylovorus* DSM 20531
M624A	SAMN31135175	112	1 995 845	12	57 998	37.84	98.60	*Lactobacillus amylovorus* DSM 20531
M668A	SAMN31135176	60	1 965 416	4	172 888	37.94	96.95	*Lactobacillus amylovorus* DSM 20531
M696A	SAMN31135177	126	1 950 594	16	36 990	37.95	98.76	*Lactobacillus amylovorus* DSM 20531
M494A	SAMN35847937	109	2 072 636	15	41 742	32.53	98.51	*Ligilactobacillus salivarius* DSM 20555
M698A	SAMN35847938	93	2 059 459	12	56 073	32.72	98.45	*Ligilactobacillus salivarius* DSM 20555

ANI = average nucleotide identity; GC = the percentage of either guanine (G) or cytosine (C) bases in DNA molecule; L50 = the count of the smallest number of contigs whose length sum makes up half of genome size; N50 = the minimum contig length required to cover 50 percent of the assembled genome sequence

The antimicrobial susceptibility and absence of horizontally transmissible antibiotic resistance genes in the genomes of all used *L. amylovorus* strains have been published in our previous article (Moravkova et al. 2022). These data are provided in Table 2 for the convenience of the reader. The MIC values of ten different antibiotics were obtained using the same method as for the *L. amylovorus* strains to assess the antibiotic susceptibility of *B. apri* and two *L. salivarius* strains.

**Table 2:** Distribution of minimal inhibition concentration for selected antimicrobials

Antibiotics range (mg/l)	Strain	No.	MIC values (mg/l)																
			< 0.63	< 0.125	0.125	0.25	0.5	1	2	4	8	16	32	64	128	> 128	256	512	1 024
Ampicillin (≤ 0.125–≥ 16)	*B. apri*	1		1															
	*L. amylovorus*	5					4	1											
	*L. salivarius*	2						2											
Streptomycin (≤ 2–≥ 256)	*B. apri*	1															1		
	*L. amylovorus*	5								1	4								
	*L. salivarius*	2															2		
Tetracycline (≤ 0.5–≥ 64)	*B. apri*	1							1										
	*L. amylovorus*	5							2	2	1								
	*L. salivarius*	2									1	1							
Erythromycin (≤ 0.063–≥ 8)	*B. apri*	1			1														
	*L. amylovorus*	5			1	4													
	*L. salivarius*	2						2											
Clindamycin (≤ 0.063–≥ 8)	*B. apri*	1			1														
	*L. amylovorus*	5				1	1	2			1								
	*L. salivarius*	2	1		1														
Vancomycin (≤ 0.25–≥ 32)	*B. apri*	1					1												
	*L. amylovorus*	5					2	3											
	*L. salivarius*	2									2								
Chloramphenicol (≤ 0.25–≥ 32)	*B. apri*	1							1										
	*L. amylovorus*	5								3	2								
	*L. salivarius*	2									2								
Kanamycin (≤ 16–≥ 2 050)	*B. apri*	1																	1
	*L. amylovorus*	5										2	1	1	1				
	*L. salivarius*	2															1	1	
Gentamicin (≤ 0.125–≥ 512)	*B. apri*	1															1		
	*L. amylovorus*	5					1		3		1								
	*L. salivarius*	2											2						
Ciprofloxacin (≤ 0.125–≥ 128)	*B. apri*	1												1					
	*L. amylovorus*	5												1	2	2			
	*L. salivarius*	2								1	1								

The gray zones represent values higher than the cut-off values for the *Lactobacillus acidophilus* group (used for *Lactobacillus amylovorus* strains), for *Lactobacillus* facultative heterofermentative (used for *Ligilactobacillus salivarius*), and for *Bifidobacterium* (used for *Bifidobacterium apri*) according to the guidance on the characterisation of microorganisms used as feed additives or production organisms (EFSA et al. 2018). The cut-off value of ciprofloxacin and kanamycin in the case of *Bifidobacterium* is not known

*B. apri* = *Bifidobacterium apri; L. amylovorus* = *Lactobacillus amylovorus; L. salivarius* = *Ligilactobacillus salivarius*; MIC = minimal inhibitory concentratio

*B. apri* exhibited MIC values above the established cut-off values for streptomycin (MIC 256 mg/ml, cut-off 128 mg/ml) and gentamicin (MIC 256 mg/l, cut-off 64 mg/l).

Resistance to streptomycin, tetracycline, chloramphenicol, kanamycin, and gentamycin was observed in the *L. salivarius* isolates (Table 2). Even though the mentioned isolates showed resistance to the antimicrobials used, whole-genome sequencing analyses using four curated databases (CARD, ResFinder, Argannot, and Megares) did not reveal any horizontally transferred resistance genes in the case of the genera *Lactobacillus* and *Ligilactobacillus*. Genome analysis of isolate 115B (*B. apri*) revealed only the presence of the genes *rpoB* and \*ileS*, which are not transferable via mobile genetic elements. β-haemolysis activity was not detected in any isolate (data not shown).

### Antimicrobial activity

Antimicrobial activity against enteropathogenic *E. coli*, *S.* Typhimurium and *Y. enterocolitica* strains was detected in *L. amylovorus* and *L. salivarius* strains, but not in the *B. apri* strain (Table 3). A high level of antimicrobial activity against all five tested pathogens, as evidenced by large inhibition zones, was observed in M494A (*L. salivarius*). *L. salivarius* strain M668A exhibited a significant antimicrobial activity against four tested pathogens, but not against *E. coli* 974. Similarly, *L. salivarius* strain M698 showed a high level of antimicrobial activity against *S.* Typhimurium and *Y. enterocolitica*, but a weaker antimicrobial activity against two of the three tested *E. coli* strains. Among the *L. amylovorus* representatives, a high antimicrobial impact was observed in the M696A isolate, inhibiting all tested pathogens except for *E. coli* 974, while M624A exhibited strong antimicrobial activity against *S.* Typhimurium, *E. coli* 971 and *E. coli* 973. Conversely, *L. amylovorus* strains M597AA and 597B showed only weak or no antimicrobial activity, particularly against *E. coli* strains. No antimicrobial activity was detected against any of the five tested pathogens in the case of isolate 115B, belonging to *B. apri*.

**Table 3:** Antimicrobial activity against pathogenic strains, production of exopolysaccharides, and presence of genes for bacteriocin production in selected strains

Strain	Identification	Antimicrobial activity					EPs	Genes for bacteriocin production
		*E. coli* 971	*E. coli* 973	*E. coli* 974	*S.* Typhimu-rium	*Y. entero-colitica*		
115B	*B. apri*	–	–	–	–	–	+	–
								
M597AA	*L. amylovorus*	+	–	–	+	+	+	helveticin J, enterolysin A, lanthipeptides class I and IV or class I
M597B	*L. amylovorus*	+	++	–	++	+	–	
M624A	*L. amylovorus*	++	+++	–	+++	–	+	
M696A	*L. amylovorus*	++	+	–	+	+++	+	
M668A	*L. amylovorus*	+	+	+	+	-	–	
								
M494A	*L. salivarius*	+	+++	++	+++	+++	–	salivaricin P, enterolysin A
M698A	*L. salivarius*	++	+	–	+++	+++	–	

– = no zone of strain growth inhibition; + = the diameter of the growth inhibition was 1 to 3 mm; ++ = the diameter of the growth inhibition was 3 to 5 mm; +++ = the diameter of the growth inhibition was more than 5 mm; *B. apri* = *Bifidobacterium apri*; EPs = exopolysaccharides; *E. coli* = *Escherichia coli*; *L. amylovorus* = *Lactobacillus amylovorus*; *L. salivarius* = *Ligilactobacillus salivarius*; *S.* Typhimurium = *Salmonella* Typhimurium; *Y. enterocolitica* = *Yersinia enterocolitica*

### Exopolysaccharide production

The production of EPs was detected visually when some isolates formed a “ropy” culture on Petri plates (Table 3). Among the examined isolates, three out of four *L. amylovorus* strains (M597AA, M624A, and M696A) as well as the *B. apri* strain (115B) were found to produce EPs.

### Bacteriocin production

The potential for bacteriocin production was evaluated using genome sequence analysis using a BLAST search in the BAGEL4 database. This approach confirmed the presence of genetic elements responsible for encoding two types of bacteriocins within the genomes of *L. salivarius* (enterolysin A and salivaricin P) and *L. amylovorus* (enterolysin A and helveticin J) (Table 3).

**Figure 1:**
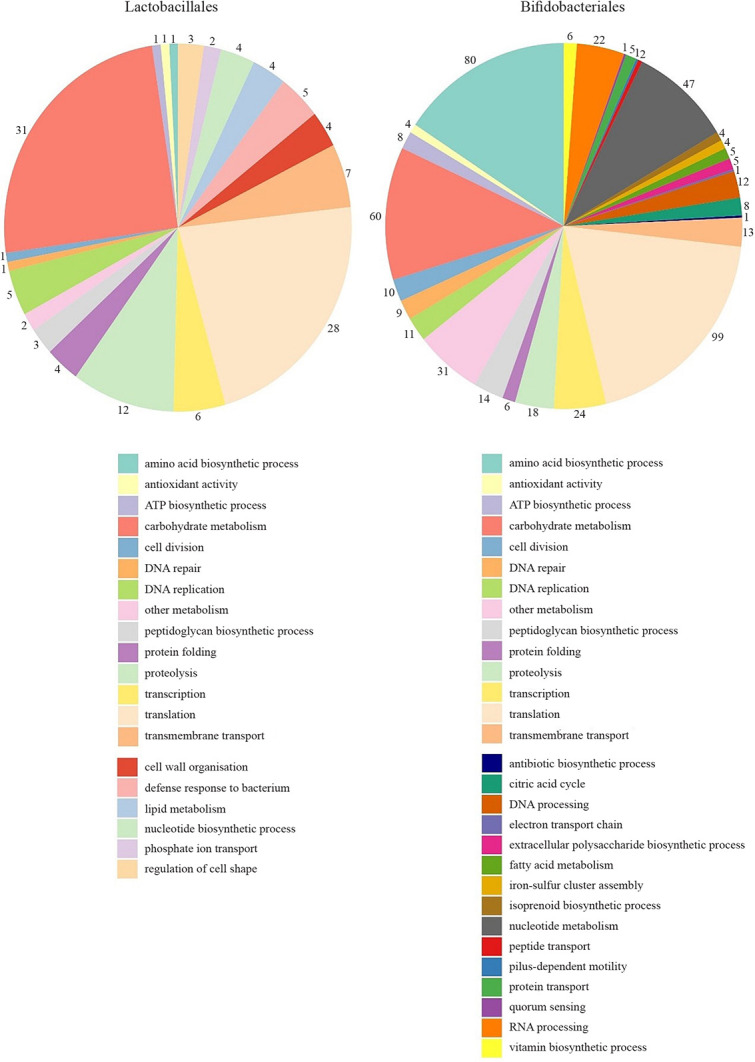
Proteins identified using mass spectrometry from bacterial supernatants and their associated biological processes according to gene ontology terms. Not all identified proteins are annotated for biological processes in a database (see above)

The presence of bacteriocins was also determined at the protein level in the supernatants obtained from all isolates studied. Out of 711 well-identified proteins (with false discovery rate (FDR) < 0.01 with at least 2 unique peptides per protein) belonging to the order *Bifidobacteriales* (*B. apri*), 511 proteins were successfully annotated. In supernatants originating from *Lactobacillales* strains (the genera *Lactobacillus* and *Ligilactobacillus*), 291 proteins were identified with 125 of them having known functions. In comparison with *Lactobacillales*, *Bifidobacteriales* contain a wide range of proteins with different functions such as “antibiotic biosynthetic process”, “extracellular polysaccharide biosynthetic process”, “quorum sensing” and “vitamin biosynthetic process”. However, none of the detected proteins from *Bifidobacteriales* supernatants participate in a “defense response to bacterium” according to gene ontology (GO) terms. Five “defense response to bacterium” proteins were identified in supernatants derived from *Lactobacillales* cultures (Figure 1).

**Figure 2:**
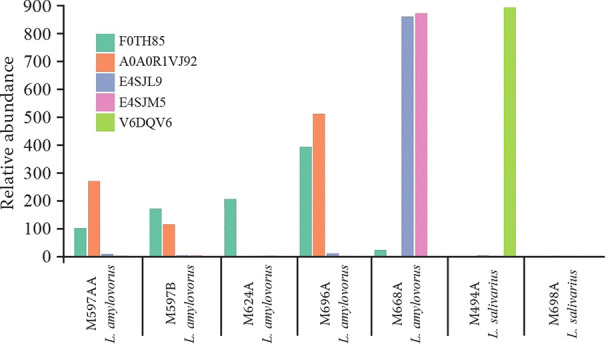
Relative abundance of proteins identified in supernatants of the order *Lactobacillales* involved in a “defense response to bacterium” biological process A0A0R1VJ92 = Bacteriocin helveticin (*L. kitasatonis*); E4SJL9 = Bacteriocin helveticin (*L. amylovorus*); E4SJM5 = Bacteriocin helveticin J (*L. amylovorus*); F0TH85 = Bacteriocin immunity protein (*L. amylovorus*); *L. amylovorus* = *Lactobacillus amylovorus*; *L. salivarius* = *Ligilactobacillus salivarius*; V6DQV6 = nonfunctional salivaricin B (*L. salivarius*)

These bacteriocins were helveticin (E4SJL9 and A0A0R1VJ92), helveticin J (E4SJM5) and bacteriocin immunity protein (F0TH85) for *Lactobacillus amylovorus* strains and nonfunctional salivaricin B (V6DQV6) for *Ligilactobacillus salivarius* strains. Their relative abundances in each isolate of the order *Lactobacillales* are shown in Figure 2. Protein sequence identity was 36% and 37% between helveticin J (E4SJM5) and both helveticin proteins (E4SJL9 and A0A0R1VJ92, respectively). The bacteriocin helveticin had a different amino acid sequence in isolates M597AA, M597B, and M696A as compared to helveticin found in the M668A isolate. Since the latter protein belonged to *L. amylovorus*, the other helveticin protein originated from a *Lactobacillus kitasatonis* strain. The sequence identity of these two proteins was 66%.

### Anti-inflammatory properties of the strains

**Figure 3:**
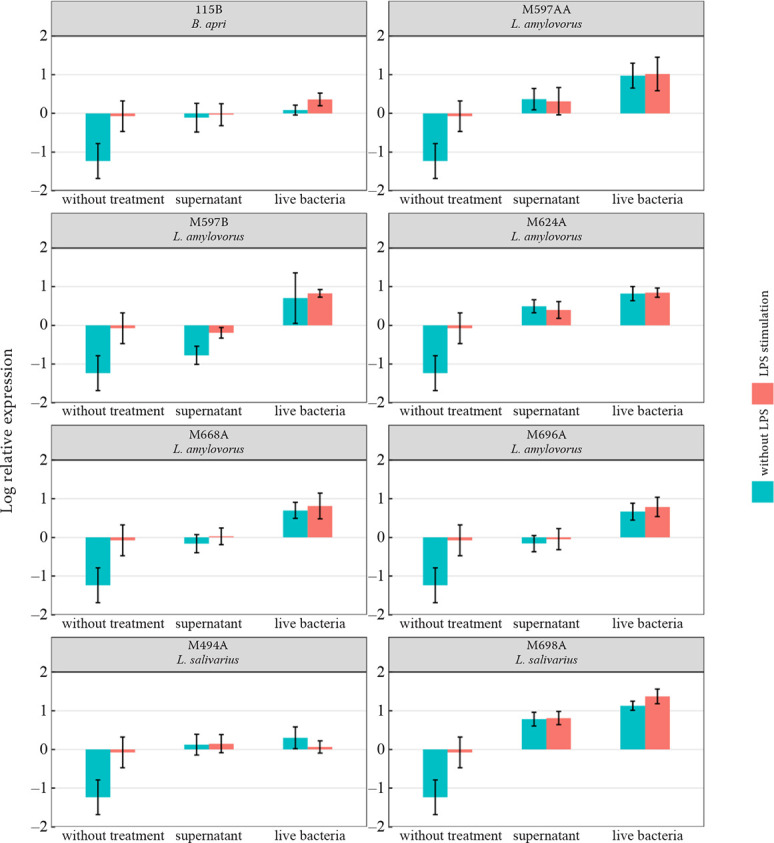
Log mRNA relative expression for interleukin-10 by monocyte-derived macrophages after 6-hour treatment with or without supernatants of live bacteria and stimulation with LPS or left without LPS stimulation. The data were logarithmised and the geometric mean was calculated. The error bars represent the standard deviation of the geometric mean. A two-factor analysis of variance (ANOVA) was conducted to analyse the data *B. apri* = *Bifidobacterium apri*; *L. amylovorus* = *Lactobacillus amylovorus*; *L. salivarius* = *Ligilactobacillus salivarius*; LPS = lipopolysaccharides

Analysis of mRNA expression revealed that macrophages responded to LPS stimulation with an increase in interleukin-10 (IL-10) expression (Figure 3). Macrophages exhibited a slight increase in IL-10 mRNA expression when treated with 10% of the supernatants and a significantly higher expression when treated with washed live bacterial cells across all tested probiotic strains. Furthermore, treatment with live bacterial cells, and to a lesser extent with supernatants, increased the IL-10 mRNA expression in macrophages, even following an LPS stimulation.

## DISCUSSION

Probiotics are live microorganisms that have beneficial effects when consumed in adequate quantities. There are several ways in which probiotics can positively influence gut well-being. These include enhancement of the epithelial barrier, inhibition of pathogen adhesion, competitive exclusion of pathogenic microorganisms, production of antimicrobial substances, and modulation of the immune system (for a review see Yousefi et al. 2019).

The bacterial strains isolated from the gut content of wild boars and tested in this study were identified as *L. amylovorus*, *L. salivarius* and *B. apri*, *L. amylovorus* and *L. salivarius* have previously been reported to have been isolated from the GIT of domestic pigs and wild boars (Bravo et al. 2019; Shen et al. 2022), whereas *B. apri* is a newly discovered species isolated from the GIT of wild boars (Pechar et al. 2017). The safety and probiotic potential, including the production of antimicrobial compounds or bacteriocins, as well as the anti-inflammatory properties of these isolates were studied using an *in vitro* approach.

Although *B. apri* showed a MIC above the established cut-off values for streptomycin (MIC 256 mg/ml, cut-off 128 mg/ml) and gentamicin (MIC 256 mg/l, cut-off 64 mg/l), it is worth noting that *Bifidobacterium* strains are generally known for their high MICs for these two antibiotics. It has been suggested that *Bifidobacterium* resistance to both antibiotics is intrinsic (Gueimonde et al. 2013; Kim et al. 2018). In addition, the genome of *B. apri* contains two genes corresponding to the intrinsic resistance to rifamycin (*rpoB* and *ileS*) (Lokesh et al. 2018) and mupirocin (Gueimonde et al. 2013). It can, therefore, be assumed that *B. apri* is a safe probiotic capable of surviving antibiotic treatment and preventing gut dysbiosis during antibiotic therapy.

Although both *L. amylovorus* and *L. salivarius* isolates exhibited phenotypic resistance to the tested antibiotics, subsequent WGS analysis did not reveal any horizontally transferred resistance genes. The susceptibility of *L. amylovorus* isolates from wild boar GIT to antibiotics was discussed in our previous study (Moravkova et al. 2022). Conversely, *in vitro* susceptibility testing of *L. salivarius* to relevant antibiotic agents has been documented in only a few studies, each with a limited number of strains. For instance, Yeo et al. (2016) analysed susceptibility to seven antibiotics in five *L. salivarius* strains isolated from pig faeces. They observed an increased MIC to streptomycin, gentamicin, and vancomycin in a range of 128–512 mg/l, 64–128 mg/l and > 512 mg/l, respectively. A broader investigation by Dec et al. (2020) explored the phenotypic and genotypic antimicrobial resistance profiles of 16 faecal strains of *L. salivarius* from domesticated pigeons. Similarly to our findings, they reported increased resistance to streptomycin and kanamycin, with MICs ranging from 32 mg/l to 256 mg/l and 256 mg/l to 512 mg/l, respectively. Additionally, they demonstrated the absence of genes associated with resistance to aminoglycosides. These results suggest that antibiotic resistance in *L. salivarius* is probably due to chromosomal mutation or other mechanisms and that *L. salivarius* strains present a low risk for the horizontal spread of genes potentially involved in antibiotic resistance. Intrinsic resistance in *Lactobacillus* spp. has been documented against vancomycin, aminoglycosides, ciprofloxacin, and trimethoprim (Campedelli et al. 2018). However, lactobacilli are generally susceptible to penicillins, β-lactams, chloramphenicol, tetracycline, linezolid, and quinupristin/dalfopristin (Abriouel et al. 2015). Similarly, intrinsic resistance in *Bifidobacterium* has been noted against muciprocin. *Bifidobacteria* are usually susceptible to macrolides, chloramphenicol, β-lactams, and vancomycin (Gueimonde et al. 2013). Since none of the investigated isolates carried transferable genetic elements for antibiotic resistance in their genomes, while at the same time not exhibiting β-haemolytic activity, these strains are considered safe from the perspective of biosecurity.

In addition to biological safety, other probiotic characteristics are essential in selecting probiotic bacteria. For instance, the production of exopolysaccharides (EPs) in probiotic bacteria can contribute to their survival, colonization, immunomodulatory effects, and promoting healthy gut microbiota in the host (Angelin and Kavitha 2020). In our study, three out of four tested *L. amylovorus* strains, as well as the *B. apri* strain, showed production of EPs consistent with findings in the literature in which EPs production was observed in lactobacilli and bifidobacteria (Chen et al. 2014; Yuan et al. 2021).

Additionally, the ability of probiotic bacteria to exhibit antimicrobial activity is another important characteristic. The mechanisms of antimicrobial activity in bacteria are numerous. Apart from the application of the principle of competitive exclusion or acidification of the gut environment, an important mechanism of probiotic bacteria is the production of bacteriocins – antimicrobial peptides – produced as a defense mechanism by bacteria to outcompete others in their environment. The strains of *L. amylovorus* and *L. salivarius* tested in our study were able to inhibit the growth of enteropathogenic *E. coli*, *S.* Typhimurium and *Y. enterocolitica*, which are common pathogens of pigs (Weber et al. 2015; Chlebicz and Slizewska 2018). The antibacterial activity of both *L. amylovorus* and *L. salivarius* has been repeatedly demonstrated *in vitro* against *E. coli* and *Salmonella* strains (Adetoye et al. 2018; Shen et al. 2022).

In our study, however, *B. apri* did not exhibit any antimicrobial activity, which contrasts with existing literature describing the antibacterial effects of bifidobacteria against specific gram-negative and gram-positive pathogens (Lim and Shin 2020). Additionally, our search using the BAGEL4 database for bacteriocin production did not reveal any genes responsible for antimicrobial activity in the genome of the *Bifidobacterium apri* isolate. Similarly, although the supernatant derived from *B. apri*, belonging to *Bifidobacteriales*, contained more identified proteins as compared to *Lactobacillales* cultures (711 vs 291), none of them were associated with the “defense response to bacterium” according to known GO terms. Conversely, the proteins identified in the supernatant of *Bifidobacteriales* exhibited a broader range of functions than those in *Lactobacillales*. Among the proteins identified in supernatants of *Bifidobacteriales* were those identified as responsible for an “antibiotic biosynthetic process”, “extracellular polysaccharide biosynthetic process”, “quorum sensing” or “vitamin biosynthetic process”, all of which could potentially contribute to the probiotic effect on the host digestive system (Pompei et al. 2007; Sabater et al. 2020; Salman et al. 2023).

On the other hand, several genes encoding bacteriocin proteins were detected in genomic sequences and confirmed to be present at a protein level in the bacterial supernatants of *L. amylovorus* and *L. salivarius* cultures. Three helveticin proteins with different amino acid sequences were found in supernatants derived from *L. amylovorus* strains. Among them, helveticin J, defined as a heat-labile bacteriocin belonging to class III of bacteriocins (Simons et al. 2020; Luo et al. 2023), has been previously identified in *L. amylovorus* strains (Collins et al. 2017; Park et al. 2023). In the case of supernatants from *L. salivarius* cultures, only one of the two tested strains produced an antibacterial protein – the nonfunctional salivaricin B (V6DQV6). Although we identified the salivaricin gene in the *L. salivarius* genome, it is not currently present in the Uniprot protein database. Based on the amino acid sequence, it shares 100% sequence identity with “Blp family class II bacteriocin”. However, analysis of *L. salivarius* strain genome sequences revealed the presence of genes encoding salivaricin P, a two-peptide bacteriocin commonly found in *L. salivarius* strains isolated from the intestines of pigs (Messaoudi et al. 2013). The presence of the gene for the bacteriocin enterolysin A was also detected in the genomes of *L. amylovorus* isolates in this study. Enterolysin A has been primarily described in enterococci (Khan et al. 2013) and *L. mucosae* (Jia et al. 2020).

Another known mechanism of how probiotic bacteria can positively influence the function of the intestine is immunomodulation. Macrophages represent innate immunity cells. One population is redistributed around specific places in the body as resident macrophages. The second population – as an inflammatory – is recruited by a chemoattractant into inflamed tissue. Macrophages can recognise corpuscular as well as soluble factors by membrane-associated pattern-recognising receptors. Both populations of macrophages play important roles in the regulation of intestinal homeostasis using the production of cytokines. One of the most important is interleukin-10 (Kole and Maloy 2014; Nguyen et al. 2021). Production of IL-10 in the small intestine is highly probably controlled by a food antigen (Kim et al. 2016). In the large intestine, however, IL-10 production is driven by microbiota (Ueda et al. 2010).

The results of our study showed that all the selected probiotic bacteria can induce the expression of IL-10 mRNA. Response to whole bacteria was significantly higher than to supernatant. Several studies demonstrated that probiotic bacteria can induce expression of mRNA for the production of the IL-10 protein. They have emphasised the role of lipoteichoic acid and its structure (Lebeer et al. 2012; Lu et al. 2022). Moreover, Engevik et al. (2021) compared the ability of cell surface components and metabolites to induce the production of IL-10, the former being more potent. These authors suggested that colonic macrophages (in contrast to colonic dendritic cells) constitutively produce IL-10 and the production is responsive for unresponsiveness to LPS stimulation. The role of commensal bacteria in these phenomena was proven by the fact that germ-free mice produced less IL-10 and had a higher response to LPS exposure. In line with this finding, our study also showed that selected probiotic bacteria can increase IL-10 production even after LPS stimulation, and cell surface structures were more potent than supernatants containing bacterial metabolites.

In this study, we isolated and selected eight potential probiotic strains originating from the digestive tract of wild boars. These isolates were identified as *Bifidobacterium apri*, *Lactobacillus amylovorus* and *Ligilactobacillus salivarius*. Our primary objective was to analyse these isolates *in vitro* for their biosecurity, antimicrobial activity, production of extracellular components, and their ability to modulate immune responses in host cells.

None of the selected isolates were found to carry transferable antibiotic resistance genes or exhibit β-haemolytic activity, suggesting their biological safety. Furthermore, all strains tested in this study exhibited the combination of probiotic properties *in vitro*.

Specifically, the isolates were capable of inhibiting the growth of selected pathogens, producing exopolysaccharides and bacteriocins, and stimulating the production of the anti-inflammatory cytokine interleukin-10 in porcine macrophages. These characteristics make the aforementioned strains, originating from the gastrointestinal tract of wild boars, promising candidates for use as probiotics in piglets.
